# A genome-wide scan for signatures of selection in Chinese indigenous and commercial pig breeds

**DOI:** 10.1186/1471-2156-15-7

**Published:** 2014-01-15

**Authors:** Songbai Yang, Xiuling Li, Kui Li, Bin Fan, Zhonglin Tang

**Affiliations:** 1 Key Laboratory of Farm Animal Genetic Resources and Germplasm Innovation of Ministry of Agriculture, Institute of Animal Science, Chinese Academy of Agricultural Sciences, Beijing 100193, P.R. China; 2 Key Laboratory of Agricultural Animal Genetics, Breeding and Reproduction, Ministry of Education & College of Animal Science and Technology, Huazhong Agricultural University, Wuhan 430070, P.R. China

**Keywords:** Pig, F-statistics, Breed, Positive selection, Network analysis

## Abstract

**Background:**

Modern breeding and artificial selection play critical roles in pig domestication and shape the genetic variation of different breeds. China has many indigenous pig breeds with various characteristics in morphology and production performance that differ from those of foreign commercial pig breeds. However, the signatures of selection on genes implying for economic traits between Chinese indigenous and commercial pigs have been poorly understood.

**Results:**

We identified footprints of positive selection at the whole genome level, comprising 44,652 SNPs genotyped in six Chinese indigenous pig breeds, one developed breed and two commercial breeds. An empirical genome-wide distribution of Fst (F-statistics) was constructed based on estimations of Fst for each SNP across these nine breeds. We detected selection at the genome level using the High-Fst outlier method and found that 81 candidate genes show high evidence of positive selection. Furthermore, the results of network analyses showed that the genes that displayed evidence of positive selection were mainly involved in the development of tissues and organs, and the immune response. In addition, we calculated the pairwise Fst between Chinese indigenous and commercial breeds (CHN VS EURO) and between Northern and Southern Chinese indigenous breeds (Northern VS Southern). The IGF1R and ESR1 genes showed evidence of positive selection in the CHN VS EURO and Northern VS Southern groups, respectively.

**Conclusions:**

In this study, we first identified the genomic regions that showed evidences of selection between Chinese indigenous and commercial pig breeds using the High-Fst outlier method. These regions were found to be involved in the development of tissues and organs, the immune response, growth and litter size. The results of this study provide new insights into understanding the genetic variation and domestication in pigs.

## Background

Pigs and humans have interacted for approximately 10,000 years, and as a major protein source for humans, the pig is one of the most important domestic animals [1]. Domestic pigs originated from the Eurasian wild boar (*Sus scrofa*) approximately 9000 years ago. European and Asian pigs were domesticated independently and introgression of the Asian domestic pig into the European pig occurred after domestication [2,3]. Most of these breeds (especially commercial breeds) have been subjected to strong artificial selection to improve pork productivity. However, different breeds show large differences in morphology and production performance due to various breeding objectives, selection systems and rearing environments; nevertheless, very little is known on the molecular mechanisms of artificial selection on pigs.

The development of high-throughput sequencing and genotyping technologies makes it possible to investigate the selective pressures of various domestic animal species at the genomic level and to identify candidate genes associated with economic traits in order to better understand the mechanisms of adaptive evolution. For example, several important genes relevant to reproduction and growth such as GHR and MC1R have been identified in cattle [4-8], and Flori *et al*. [6] implemented a network analysis for detected genes that have been putatively subjected to selection. Akey *et al*. [9] identified 155 regions in the canine genome that have likely been subjected to strong artificial selection, including the HAS2 gene, which is involved in skin wrinkling. The thyroid stimulating hormone receptor (TSHR) gene was identified as having undergone strong artificial selection in domestic chickens [10]. The above studies used several types of approaches that were based on either the allele frequency spectrum or the properties of haplotype segregation in populations to detect signals of recent positive selection on a genome-wide scale [8]. For example, Fst (a measure of population differentiation) provides an estimate of the genetic variability between populations: a locus that shows significantly high Fst statistics compared with other loci provides evidence for positive selection [11]. Akey *et al*. [12] suggested that the loci in the tails of the empirical distribution of Fst be used as candidate targets of selection. Another method of identifying loci under selection is the EHH (Extended Haplotype Homozygosity) test [13], which identifies the genome regions that have unusually high LD and allele frequency.

The advent of the Illumina Porcine SNP60 BeadChip [14] allows for the investigation of selective pressure at the genome-wide level in pigs. Melanocortin receptor 1 gene (MC1R) was identified as an artificial selection gene related to coat colour in Chinese domestic pigs [15]. A missense mutation in the PPARD gene had an effect on the ear size of the pigs [16]. China has a number of indigenous pig breeds, most of which are fat-type and low degree of nurturing breeds. Therefore, using Chinese indigenous breeds would be a better way to obtain meaningful signatures of selection on genes implying for economic traits in the pig at genomic level. Therefore, the objective of this study was to identify regions subjected to recent artificial selection using a genome scan for SNP differences. The findings will contribute to the construction of a positive selection map, which could help us to understand the recent breeding history of different pig breeds. Our results will also facilitate the identification of candidate genes that are important for economic traits for breeding practices.

## Results

### Population structure and genome-wide distribution of Fst

**Figure 1:**
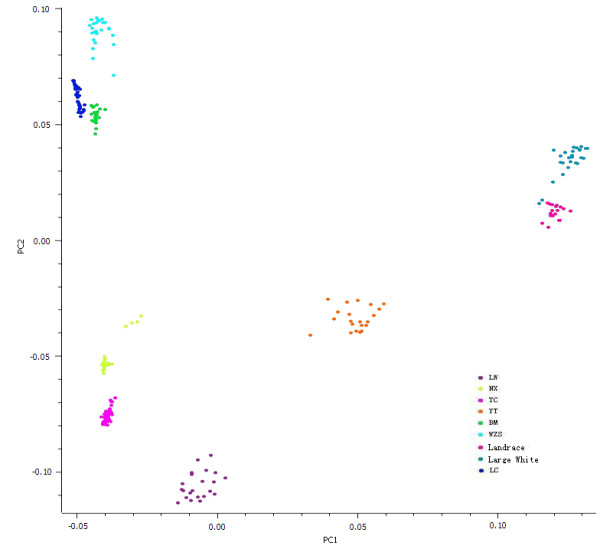
**Principal component analysis results based on whole genome SNP data.** (235 individuals, 44,652 SNPs). Individuals are plotted according to their coordinates on the biplot of PC1 versus PC2. Breed abbreviations are described in Materials and Methods.

To examine the genetic structure of the studied populations, the principle component analysis (PCA) was conducted based on all available SNP information. As shown in Figure 1, the first two components accounted for 42.43% and 8.94% of the variation, respectively. The Luchuan, Bama and Wuzhishan pigs were clustered closely, as were the Ningxiang and Tongcheng pigs and the Large White and Landrace pigs, while the Yutai and Laiwu were more distant from the other pig breeds.

**Figure 2:**
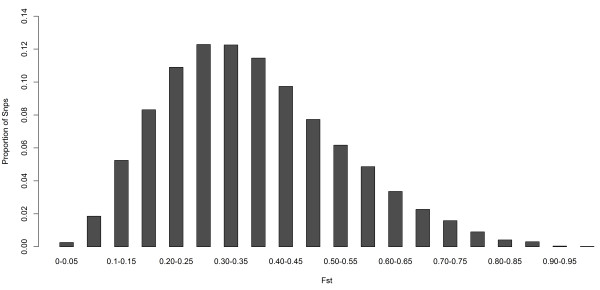
Genome-wide distribution of Fst in ALLPOP group.

**Figure 3:**
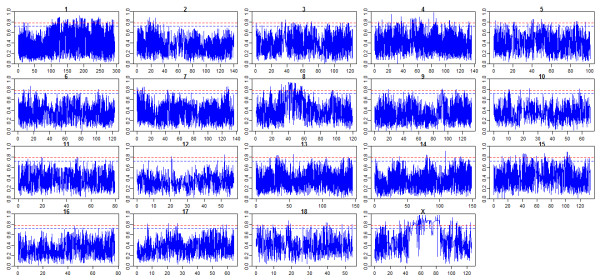
**Fst for each SNP as a function of chromosome position in Mb in the ALLPOP group.** The red dashed line corresponds to the 99% threshold on the corresponding empirical distributions, and the blue indicates the 97.5% threshold.

We constructed the empirical genome-wide distribution of global Fst estimates based on 44,652 SNPs of the nine breeds (ALLPOP) in order to examine the inter-locus variation in allele frequencies (Figure 2). The average Fst of these loci was 0.3717 with standard deviation 0.16. Local environmental adaptation and artificial selection can change the allele frequencies of specific loci: the frequency of advantageous alleles at the selected loci will increase, leading to a higher than expected level of population differentiation (Fst) [12]. The genome-wide distribution of Fst revealed selection in the pig genome. To identify specific genomic regions containing signatures of selection, we constructed a chromosomal distribution of Fst as a function of chromosome position. As shown in Figure 3, the sex chromosomes have a smaller effective population size compared to the autosomes, which makes them more sensitive to demographic events and/or natural selection [12]. As a result, there was an unexpectedly high Fst level on the physical position 40-80 M of the X chromosome. Taking into account the PCA analysis results, the pairwise Fst between Chinese indigenous and European commercial breeds were calculated by merging Chinese indigenous breeds and commercial breeds into two groups (CHN VS EURO). In addition, the pairwise Fst between Northern (LW pigs) and Southern Chinese indigenous breeds was calculated by merging LC, WZS and BM into one group (Northern VS Southern).

### Candidate genes under selection

To identify loci subjected to selection, we focused on the high-Fst outlier method corresponding to the distribution of Fst. According to the empirical distribution of Fst estimates, we selected the high-Fst outlier SNPs that corresponded to the upper 1% of the distribution as the loci under selection. In the ALLPOP group, a total of 446 SNPs were determined to be subjected to natural or artificial selection following this criteria, and these SNPs were from a total of 81 candidate genes (Additional file 1: Table S1). In addition, a total of 84 and 79 candidate genes were identified in the CHN VS EURO group and the Northern VS Southern group, respectively (Additional file 2: Table S2 and Additional file 3: Table S3). Several candidate genes contain contiguous outlier-Fst SNPs; for example, the transient receptor potential three (TRPM3) gene contains five contiguous SNPs with Fst values that are consistently high, and the nuclear envelope spectrin repeat 2 (Nesprin-2) gene contained two outlier-Fst SNPs in the ALLPOP group.

### Functional analysis of candidate genes under selection

**Figure 4:**
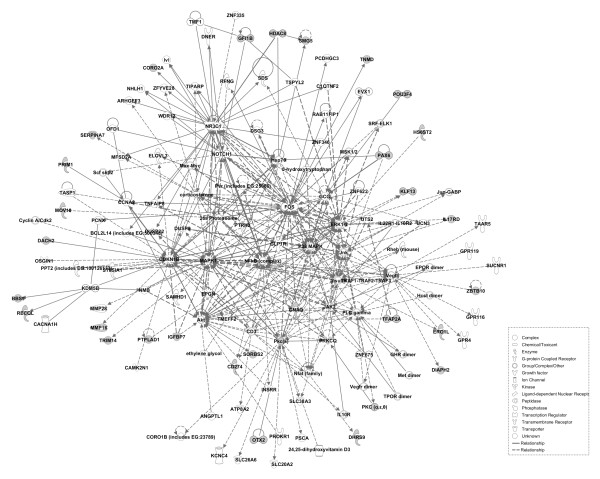
**Representation of the gene network N.** Symbols corresponding to genes under selection are colored in grey.

**Figure 5:**
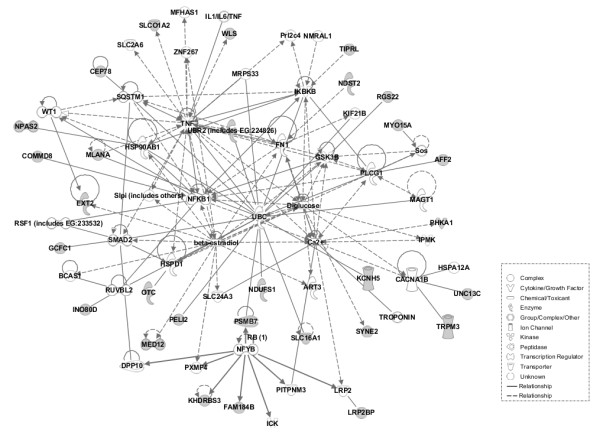
**Representation of the gene network N1.** Symbols corresponding to genes under selection are colored in grey.

Based on a system biology approach, we carried out network analysis using IPA software to identify the critical physiological pathways of the genes harbouring footprints of positive selection. The pig breeds selected have obvious differences in both morphology and performance. The Large White and Landrace pigs are well-known commercial breeds with high meat productivity, fast growth, and high adaptability; however, Chinese indigenous breeds vary in morphological and performance phenotypes and in local environmental suitability. For example, the Bama and Wuzhishan pigs from Southern China have a small body size, while the Laiwu pigs from Northern China are larger. First, 75 out of 81 genes in the ALLPOP group were mapped to the IPA database, and then three significance networks, namely N1, N2 and N3, were constructed. N2 and N3 were interconnected and further merged into a single network (N). Networks N and N1 are represented in Figures 4 and 5, respectively. The main hubs of the N network contained genes encoding protein kinases (Akt, Erk, Mapk, JAK2, PKC), transcription factors (NFκB, FOS), and several other signalling molecules (Insulin, CDKN1B, NR3C1, Vegf). The N network contained 32 candidate genes under selection (CD274, DHRS9, DIAPH2, ERO1L, GLP2R, GNAQ, HDAC8, HS6ST2, IGFBP7, IL17RD, JAK2, KLF13, MOV10, OTX2, PAX6, PPKCQ, PTPLAD1, SMG5, SORBS2, TFAP2A, ZBTB10, BBS9, CORO2A, DACH2, GFI1B, MMP16, POU3F4, PRIM1, RECQL, SERPINA7, TNMD, TRIM14), and the N1 network contained 29 candidate genes (AFF2, CEP78, COMMD8, EXT2, FAM184B, GCFC1, INO80D, KCNH5, KHDRBS3, LRP2BP, MED12, MLANA, MYO15A, NDST2, NDUFS1, NPAS2, OTC, PELI2, PHKA1, PSMB7, RGS22, SLC16A1, SLCO1A2, SYNE2, TIPRL, TRPM2, UBR2, UNC13C, WLS). These molecules were mainly involved in morphology, cellular function and maintenance, the cell cycle and signaling. The main hubs of the network contained IGF1R, JAK2 and calmodulin in the CHN VS EURO group (Additional file 4: Figure S1) and ESR1,PKC and insulin in Northern VS Southern group (Additional file 5: Figure S2).

## Discussion

In this study, the population structure of the nine pig breeds was analyzed, and the PCA results showed that most of the individuals could be classified into their breeds using the first and second eigenvectors (Figure 1). As with other livestock species such as cattle and sheep [17,18], the combination of PC1 and PC2 separated individuals according to their geographic origin: of all the studied breeds, the indigenous breeds of Southern China (Wuzhishan, Bama, Luchuan) clustered together, as did the breeds of Central China (Nixiang, Tongcheng), the Northern Chinese breed (Laiwu) and a developed breed (Yutai) formed a separate single cluster, and two commercial breeds, the Large White and Landrace, formed a distinct cluster. There was almost no overlap between the nine different pig breeds. This opens the possibility that an informative SNP panel can be used to assign parentage, which has proven successful in cattle [19].

Pigs have been undergoing selection to enhance performance and productivity during domestication and breed formation. In the present study, global and pairwise Fst was utilized to detect genetic selection in Chinese indigenous and commercial pig breeds. First, the ALLPOP group showed evidence of selection on chromosomes 8 (Figure 3). We identified selection near *KIT*, which can affect coat colour in pigs when mutated [20] and also shows high evidence of selection in sheep [18]. In addition, as shown in Figures 4 and 5, the N network contained several hubs involved with physiological signaling molecules (NFκB, MAPK, ERK). These data indicate that these genes participate in the basic physiological processes, and the N network contained hubs (TNF and beta-estradiol) showing that the genes under selection are involved in the immune response and reproductive traits. The POU3F4 and OTX2 genes are important for the development of cochlea, and mutants of these two genes in mice cause developmental defects in the inner ear [21,22]. In mouse embryonic stem cells, the mutant zinc-finger proto-oncogene GFI1B gene decreases erythropoiesis of embryonic stem cells [23]. The PAX6 gene is necessary and sufficient to trigger the cascade of events required for eye formation [24]. The PAX6 and OTX2 genes also play important role in the development of the body axis [25,26]. In addition, several identified molecules are involved in the development of organs. The GNAQ gene can regulate cardiac growth and development, and mice lacking both GNAQ and GNA11 [Gaq(−/−); Ga11(−/−)] died at embryonic day 11 due to cardiomyocyte hypoplasia [27]. The TFAP2A gene is a critical transcription factor for epidermal differentiation and interacts with notch signaling molecules [28].

Several candidate genes are also involved in molecular transportation in the ALLPOP group. For example, the SLOC1A2 and SERPINA7 genes can increase the transport of thyroid hormone in the serum [29,30]. The SLC16A1 gene plays an important role in the transport of mevalonate and ketone bodies [31,32]. Among the candidate genes under selection, some are associated with genetic disorders and cancer in humans. GWAS results showed that a SNP substitution mutation of BBS9 was associated with amyotrophic lateral sclerosis [33]; furthermore, BANF2, SNX25, SAMD12 and GPR177 were associated with Crohn’s disease [34], and inflammatory bowel disease was associated with the upregulation of human CD274 at the cell surface from macrophage-derived dendritic cells of the inflamed colon [35]. In addition, three genes (DIAPH2, AFF2, POF1B) were involved in functions related to premature ovarian failure [36,37]. In the CHN VS EURO group, the network hubs are gathered at the centre with the IGF1R (Insulin-like growth factor 1 receptor) gene (Additional file 4: Figure S1), which is necessary for normal growth. IGF1R null mice die at birth of respiratory failure and exhibit only 45% of the body weight of their wild-type littermates [38]. European commercial pig breeds grow faster in contrast with Chinese indigenous breeds. In addition, the IGF1R gene also showed a strong signature of selection in European domestic pigs [39]. Interestingly, one of the most critical signalling molecules, JAK2, showed high evidence of positive selection both in the ALLPOP and the CHN VS EURO groups (Figure 4 and Additional file 4: Figure S1). JAK2 is an essential gene in mammals and participates in a variety of biological processes; the loss of JAK2 is lethal [40]. JAK2 is also involved in the immune response [41], and it has been suggested that these pig breeds may have different resistance to pathogens. In the Northern VS Southern group, the central hub of the network was the ESR1 (esotrogen receptor 1) gene (Additional file 5: Figure S2). The ESR1 gene was associated with litter size in pigs and was also a candidate gene for boar fertility and sperm quality [42-44]. Laiwu pigs have a higher reproductive capacity compared with the Bama and Wuzhishan pigs. Correspondingly, the ESR1 gene showed high evidence of positive selection in our study.

The high-Fst outlier method is a powerful tool for the detection of positive selection [45]; however, the high correlation between Fst estimates when loci are in strong disequilibrium makes it difficult to determine whether the Fst at particular SNP is markedly different from the expected values [46]. We also tested the correlation of Fst between pairs of SNPs as a function of marker distances; the correlation of Fst tended to drop quickly toward 0 when SNPs were more than 300 kb apart (data not shown). Modern pig breeds had much larger average linkage disequilibrium (LD) than humans and cattle [47], therefore, the results in pigs were greater than in humans and bovines [6,12].

## Conclusions

Overall, a genome-wide scan was performed in Chinese indigenous pigs to help interpret artificial selection and adaptive evolution. We constructed population structures and genome-wide distributions of Fst. A number of genes were identified as displaying signatures of selection, and several critical physiological pathways of these genes were determined to have footprints of positive selection. Some of these genes play important roles in biological processes, which can be used to interpret the differences between these pig breeds.

## Methods

### DNA samples and SNP chip data quality control

DNA samples were obtained from 235 pigs from nine breeds from different areas of China, including six indigenous breeds: Tongcheng (TC, n = 35), Bama (BM, n = 22), Laiwu (LW, n = 23), Wuzhishan (WZS, n = 25), Ningxiang (NX, n = 24), Luchuan(LC, n = 40), two commercial breeds, Landrace (n = 18) and Large White (n = 26), and one developed breed, Yutai (YT, n = 22).

Genotyping was carried out using the Illumina Porcine SNP60 BeadChip [14], which contains a total of 62,123 SNPs. Quality control was determined using the PLINK programme [48]. A total of 8,383 unmapped markers (Based on *Sus Scrofa* Build 9.0) and 8,391 loci were filtered to exclude markers with a minor allele frequency (MAF) < 0.05. A total of 2,709 markers that were genotyped on less than 90% of all individuals were discarded from further analysis. The final data set consisted of 44,652 SNPs from nine breeds.

### Population structure and Fst estimation

Principal component analysis (PCA) based on all available SNP information was performed using the SVS7 software (Golden Helix Inc., Bozeman, MT,USA). Fst statistics across populations were estimated using the Genepop 4.1 program [49]. Fst is a measure of population differentiation, which is defined as $\mathrm{Fst}=\frac{\mathrm{MSP}-\mathrm{MSI}}{\mathrm{MSP}+\left(n_{c}-1\right)\mathrm{MSI}+n_{c}\mathrm{MSG}}$,

where *MSG*, *MSI* and *MSP* represent the mean sums of squares for gametes, individuals and populations computed by an analysis of variance, respectively, and *n*_*c*_ = (*S*_1_ − *S*_2_/*S*_1_)/(*n* − 1), where *S*_*1*_ is the total sample size, *S*_*2*_ is the sum of squared group sizes, and *n* is the number of non-empty groups.

### Identification of candidate genes under selection

Genome regions containing the high-Fst outliers corresponding to the distribution of Fst were identified as follows: for all loci, a region was considered to be a high-Fst outlier if it corresponded to the upper 1% of the empirical genome-wide distribution of Fst. A gene was regarded as being under selection if it contained unexpectedly highly differentiated SNPs among the populations. All of the high-Fst outlier loci were mapped to gene-associated regions based on the pig genome annotation (*Sus Scrofa* Build 9.0 version). An SNP was considered to be from a particular gene if it mapped to either the 5′ upstream, 5′ UTR, coding, intronic, 3′UTR, or 3′ downstream region of the gene.

### Network analysis of candidate genes

Network analysis was aimed at searching for the direct or indirect interactions between candidate molecules and the related property. The known interactions were annotated by experts according to the literature. Ingenuity Pathway Analysis (IPA) v7.0 (Ingenuity Systems Inc., USA, http://www.ingenuity.com/) was used to construct networks. We uploaded the genes being subjected to selection into this software and organized them into networks of interacting genes to identify several pathways containing important functionally related genes. This network analysis approach was similar to the described by Flori [6]. The genes that displayed evidence of selection were uploaded into IPA based on the eligible candidate genes, and IPA automatically constructed several networks that contained a limit of 70 molecules (including candidate genes).

## Competing interests

The authors declare that they have no competing interests.

## Authors’ contributions

SY and XL carried out the statistical analysis and drafted the manuscript, BF participated in the study design and manuscript preparation, and ZT and KL contributed to the data collection and genotyped all of the samples. All authors contributed to editing the article and approved the final manuscript.

## Supplementary Material

Additional file 1: Table S1 Candidate genes under selection with SNPs in high Fst in group ALLPOP. Click here for file

Additional file 2: Table S2 Candidate genes under selection with SNPs in high Fst (CHN VS EURO). Click here for file

Additional file 3: Table S3 Candidate genes under selection with SNPs in high Fst (Northern VS Southern). Click here for file

Additional file 4: Figure S1 Representation of the gene network group CHN VS EURO. Symbols corresponding to genes under selection are colored in grey. Click here for file

Additional file 5: Figure S2 Representation of the gene network group Northern VS Southern. Symbols corresponding to genes under selection are colored in grey. Click here for file

## References

[B1] Albarella U Dobney K Ervynk A Rowley-Conwy P Pigs and Humans: 10,000 Years of Interaction 2007 Oxford: Oxford University Press

[B2] Giuffra E Kijas JM Amarger V Carlborg O Jeon JT Andersson L The origin of the domestic pig: independent domestication and subsequent introgression Genetics 2000 154 4 1785 1791 10747069 10.1093/genetics/154.4.1785 PMC1461048

[B3] Groenen MA Archibald AL Uenishi H Tuggle CK Takeuchi Y Rothschild MF Rogel-Gaillard C Park C Milan D Megens HJ Analyses of pig genomes provide insight into porcine demography and evolution Nature 2012 491 7424 393 398 10.1038/nature11622 23151582 PMC3566564

[B4] Gautier M Flori L Riebler A Jaffrezic F Laloe D Gut I Moazami-Goudarzi K Foulley JL A whole genome Bayesian scan for adaptive genetic divergence in West African cattle BMC Genomics 2009 10 550 10.1186/1471-2164-10-550 19930592 PMC2784811

[B5] Gautier M Naves M Footprints of selection in the ancestral admixture of a New World Creole cattle breed Mol Ecol 2011 20 15 3128 3143 10.1111/j.1365-294X.2011.05163.x 21689193

[B6] Flori L Fritz S Jaffrezic F Boussaha M Gut I Heath S Foulley JL Gautier M The genome response to artificial selection: a case study in dairy cattle PLoS One 2009 4 8 e6595 10.1371/journal.pone.0006595 19672461 PMC2722727

[B7] Qanbari S Pimentel EC Tetens J Thaller G Lichtner P Sharifi AR Simianer H A genome-wide scan for signatures of recent selection in Holstein cattle Anim Genet 2010 41 4 377 389 20096028 10.1111/j.1365-2052.2009.02016.x

[B8] Qanbari S Gianola D Hayes B Schenkel F Miller S Moore S Thaller G Simianer H Application of site and haplotype-frequency based approaches for detecting selection signatures in cattle BMC Genomics 2011 12 1 318 10.1186/1471-2164-12-318 21679429 PMC3146955

[B9] Akey JM Ruhe AL Akey DT Wong AK Connelly CF Madeoy J Nicholas TJ Neff MW Tracking footprints of artificial selection in the dog genome Proc Natl Acad Sci U S A 2010 107 3 1160 1165 10.1073/pnas.0909918107 20080661 PMC2824266

[B10] Rubin CJ Zody MC Eriksson J Meadows JR Sherwood E Webster MT Jiang L Ingman M Sharpe T Ka S Whole-genome resequencing reveals loci under selection during chicken domestication Nature 2010 464 7288 587 591 10.1038/nature08832 20220755

[B11] Nielsen R Molecular signatures of natural selection Annu Rev Genet 2005 2005 39 197 218 10.1146/annurev.genet.39.073003.112420 16285858

[B12] Akey JM Zhang G Zhang K Jin L Shriver MD Interrogating a high-density SNP map for signatures of natural selection Genome Res 2002 12 12 1805 1814 10.1101/gr.631202 12466284 PMC187574

[B13] Sabeti PC Reich DE Higgins JM Levine HZ Richter DJ Schaffner SF Gabriel SB Platko JV Patterson NJ McDonald GJ Detecting recent positive selection in the human genome from haplotype structure Nature 2002 419 6909 832 837 10.1038/nature01140 12397357

[B14] Ramos AM Crooijmans RP Affara NA Amaral AJ Archibald AL Beever JE Bendixen C Churcher C Clark R Dehais P Design of a high density SNP genotyping assay in the pig using SNPs identified and characterized by next generation sequencing technology PLoS ONE 2009 4 8 e6524 10.1371/journal.pone.0006524 19654876 PMC2716536

[B15] Li J Yang H Li JR Li HP Ning T Pan XR Shi P Zhang YP Artificial selection of the melanocortin receptor 1 gene in Chinese domestic pigs during domestication Heredity 2010 105 3 274 281 10.1038/hdy.2009.191 20179735

[B16] Ren J Duan Y Qiao R Yao F Zhang Z Yang B Guo Y Xiao S Wei R Ouyang Z A missense mutation in PPARD causes a major QTL effect on ear size in pigs PLoS Genet 2011 7 5 e1002043 10.1371/journal.pgen.1002043 21573137 PMC3088719

[B17] Gibbs RA Taylor JF Van Tassell CP Barendse W Eversole KA Gill CA Green RD Hamernik DL Kappes SM Lien S Genome-wide survey of SNP variation uncovers the genetic structure of cattle breeds Science 2009 324 5926 528 532 19390050 10.1126/science.1167936 PMC2735092

[B18] Kijas JW Lenstra JA Hayes B Boitard S Porto Neto LR San Cristobal M Servin B McCulloch R Whan V Gietzen K Genome-wide analysis of the world’s sheep breeds reveals high levels of historic mixture and strong recent selection PLoS Biol 2012 10 2 e1001258 10.1371/journal.pbio.1001258 22346734 PMC3274507

[B19] Heaton MP Harhay GP Bennett GL Stone RT Grosse WM Casas E Keele JW Smith TP Chitko-McKown CG Laegreid WW Selection and use of SNP markers for animal identification and paternity analysis in U.S. beef cattle Mamm Genome 2002 13 5 272 281 10.1007/s00335-001-2146-3 12016516

[B20] Fontanesi L D’Alessandro E Scotti E Liotta L Crovetti A Chiofalo V Russo V Genetic heterogeneity and selection signature at the KIT gene in pigs showing different coat colours and patterns Anim Genet 2010 41 5 478 492 10.1111/j.1365-2052.2010.02054.x 20477793

[B21] Morsli H Tuorto F Choo D Postiglione MP Simeone A Wu DK Otx1 and Otx2 activities are required for the normal development of the mouse inner ear Development 1999 126 11 2335 2343 10225993 10.1242/dev.126.11.2335

[B22] Phippard D Lu L Lee D Saunders JC Crenshaw EB 3rd Targeted mutagenesis of the POU-domain gene Brn4/Pou3f4 causes developmental defects in the inner ear J Neurosci 1999 19 14 5980 5989 10407036 10.1523/JNEUROSCI.19-14-05980.1999 PMC6783103

[B23] Saleque S Cameron S Orkin SH The zinc-finger proto-oncogene Gfi-1b is essential for development of the erythroid and megakaryocytic lineages Genes Dev 2002 16 3 301 306 10.1101/gad.959102 11825872 PMC155332

[B24] Chow RL Altmann CR Lang RA Hemmati-Brivanlou A Pax6 induces ectopic eyes in a vertebrate Development 1999 126 19 4213 4222 10477290 10.1242/dev.126.19.4213

[B25] Kimura C Shen MM Takeda N Aizawa S Matsuo I Complementary functions of Otx2 and Cripto in initial patterning of mouse epiblast Dev Biol 2001 235 1 12 32 10.1006/dbio.2001.0289 11412024

[B26] Bishop KM Rubenstein JL O’Leary DD Distinct actions of Emx1, Emx2, and Pax6 in regulating the specification of areas in the developing neocortex J Neurosci 2002 22 17 7627 7638 12196586 10.1523/JNEUROSCI.22-17-07627.2002 PMC6757966

[B27] Offermanns S Zhao LP Gohla A Sarosi I Simon MI Wilkie TM Embryonic cardiomyocyte hypoplasia and craniofacial defects in G alpha q/G alpha 11-mutant mice EMBO J 1998 17 15 4304 4312 10.1093/emboj/17.15.4304 9687499 PMC1170764

[B28] Wang X Pasolli HA Williams T Fuchs E AP-2 factors act in concert with Notch to orchestrate terminal differentiation in skin epidermis J Cell Biol 2008 183 1 37 48 10.1083/jcb.200804030 18824566 PMC2557040

[B29] Vranckx R Rouaze-Romet M Savu L Mechighel P Maya M Nunez EA Regulation of rat thyroxine-binding globulin and transthyretin: studies in thyroidectomized and hypophysectomized rats given tri-iodothyronine or/and growth hormone J Endocrinol 1994 142 1 77 84 10.1677/joe.0.1420077 7964287

[B30] Xu C Li CY Kong AN Induction of phase I, II and III drug metabolism/transport by xenobiotics Arch Pharm Res 2005 28 3 249 268 10.1007/BF02977789 15832810

[B31] Pardridge WM The blood–brain barrier: bottleneck in brain drug development NeuroRx: J Am Soc Exp NeuroTherapeutics 2005 2 1 3 14 10.1602/neurorx.2.1.3 PMC539316 15717053

[B32] Kim CM Goldstein JL Brown MS cDNA cloning of MEV, a mutant protein that facilitates cellular uptake of mevalonate, and identification of the point mutation responsible for its gain of function J Biol Chem 1992 267 32 23113 23121 1429658

[B33] van Es MA Van Vught PW Blauw HM Franke L Saris CG Andersen PM Van Den Bosch L de Jong SW Van’t Slot R Birve A ITPR2 as a susceptibility gene in sporadic amyotrophic lateral sclerosis: a genome-wide association study Lancet Neurol 2007 6 10 869 877 10.1016/S1474-4422(07)70222-3 17827064

[B34] Consortium TWTCC Genome-wide association study of 14,000 cases of seven common diseases and 3,000 shared controls Nature 2007 447 7145 661 678 10.1038/nature05911 17554300 PMC2719288

[B35] Wang S Chen L Co-signaling molecules of the B7-CD28 family in positive and negative regulation of T lymphocyte responses Microbes and Infection/Institut Pasteur 2004 6 8 759 766 10.1016/j.micinf.2004.03.007 15207823

[B36] Goswami D Conway GS Premature ovarian failure Hum Reprod Update 2005 11 4 391 410 10.1093/humupd/dmi012 15919682

[B37] Lacombe A Lee H Zahed L Choucair M Muller JM Nelson SF Salameh W Vilain E Disruption of POF1B binding to nonmuscle actin filaments is associated with premature ovarian failure Am J Hum Genet 2006 79 1 113 119 10.1086/505406 16773570 PMC1474115

[B38] Liu JP Baker J Perkins AS Robertson EJ Efstratiadis A Mice carrying null mutations of the genes encoding insulin-like growth factor I (Igf-1) and type 1 IGF receptor (Igf1r) Cell 1993 75 1 59 72 8402901

[B39] Rubin CJ Megens HJ Martinez Barrio A Maqbool K Sayyab S Schwochow D Wang C Carlborg O Jern P Jorgensen CB Strong signatures of selection in the domestic pig genome Proc Natl Acad Sci U S A 2012 109 48 19529 19536 10.1073/pnas.1217149109 23151514 PMC3511700

[B40] Neubauer H Cumano A Muller M Wu H Huffstadt U Pfeffer K Jak2 deficiency defines an essential developmental checkpoint in definitive hematopoiesis Cell 1998 93 3 397 409 10.1016/S0092-8674(00)81168-X 9590174

[B41] Zhong J Yang P Muta K Dong R Marrero M Gong F Wang CY Loss of Jak2 selectively suppresses DC-mediated innate immune response and protects mice from lethal dose of LPS-induced septic shock PLoS ONE 2010 5 3 e9593 10.1371/journal.pone.0009593 20231889 PMC2834745

[B42] Munoz G Ovilo C Estelle J Silio L Fernandez A Rodriguez C Association with litter size of new polymorphisms on ESR1 and ESR2 genes in a Chinese-European pig line Genet Sel Evol 2007 39 2 195 206 10.1186/1297-9686-39-2-195 17306201 PMC2682837

[B43] Gunawan A Kaewmala K Uddin MJ Cinar MU Tesfaye D Phatsara C Tholen E Looft C Schellander K Association study and expression analysis of porcine ESR1 as a candidate gene for boar fertility and sperm quality Anim Reprod Sci 2011 128 1–4 11 21 21944540 10.1016/j.anireprosci.2011.08.008

[B44] van Rens BT de Groot PN van der Lende T The effect of estrogen receptor genotype on litter size and placental traits at term in F2 crossbred gilts Theriogenology 2002 57 6 1635 1649 10.1016/S0093-691X(02)00671-4 12035975

[B45] Karlsson S Moen T The power to detect artificial selection acting on single loci in recently domesticated species BMC Res Notes 2010 3 232 10.1186/1756-0500-3-232 20796289 PMC2942893

[B46] Holsinger KE Weir BS Genetics in geographically structured populations: defining, estimating and interpreting FST Nat Rev Genet 2009 10 9 639 650 10.1038/nrg2611 19687804 PMC4687486

[B47] Zhang C Plastow G Genomic Diversity in Pig (Sus scrofa) and its Comparison with Human and other Livestock Curr Genomics 2011 12 2 138 146 10.2174/138920211795564386 21966252 PMC3129048

[B48] Purcell S Neale B Todd-Brown K Thomas L Ferreira MA Bender D Maller J Sklar P de Bakker PI Daly MJ PLINK: a tool set for whole-genome association and population-based linkage analyses Am J Hum Genet 2007 81 3 559 575 10.1086/519795 17701901 PMC1950838

[B49] Raymond M Rousset F GENEPOP (version 1.2): population genetics software for exact tests and ecumenicism J Hered 1995 86 248 249

