# Supplementary material for: A genome-wide scan for signatures of selection in Chinese indigenous and commercial pig breeds
Source: BMC Genet. 2014 Jan 15;15:7. doi: 10.1186/1471-2156-15-7 (PMC3898232; doi:10.1186/1471-2156-15-7)
Supplement: Additional file 1: Table S1 — Candidate genes under selection with SNPs in high Fst in group ALLPOP. [file 1471-2156-15-7-S1.docx]

**Supplemental table 1: Candidate genes under selection with SNPs in high Fst in group ALLPOP.**

| SNP | Chr | Position | Fst | Gene Start (bp) | Gene End (bp) | Ensembl Gene ID | Within_Gene |
| --- | --- | --- | --- | --- | --- | --- | --- |
| ALGA0025198 | 4 | 57797738 | 0.8613 | 57783815 | 57815361 | ENSSSCG00000006155 | ZBTB10 |
| DRGA0001466 | 1 | 121936922 | 0.8739 | 121861605 | 122421065 | ENSSSCG00000004614 | UNC13C |
| ASGA0033096 | 7 | 43494969 | 0.7911 | 43401505 | 43630342 | ENSSSCG00000001641 | UBR2 |
| DRGA0002019 | 1 | 234420443 | 0.8275 | 234286063 | 234571680 | ENSSSCG00000005257 | TRPM3 |
| ALGA0008740 | 1 | 251478309 | 0.8605 | 251468742 | 251495122 | ENSSSCG00000005374 | TRIM14 |
| MARC0018104 | 19 | 75615013 | 0.8356 | 75611723 | 75635367 | ENSSSCG00000012480 | TNMD |
| ALGA0026258 | 4 | 86055343 | 0.8726 | 86049538 | 86082876 | ENSSSCG00000006301 | TIPRL |
| BGIS0007227 | 1 | 205148278 | 0.862 | 205142406 | 205148310 | ENSSSCG00000005123 | Tie-2 |
| M1GA0009495 | 7 | 7632034 | 0.8173 | 7630425 | 7648184 | ENSSSCG00000001036 | TFAP2A |
| ALGA0007467 | 1 | 202969922 | 0.862 | 202841045 | 203109516 | ENSSSCG00000005110 | SYNE2 |
| ALGA0005765 | 1 | 124600282 | 0.8758 | 124590098 | 124603484 | ENSSSCG00000004624 | SUSERK3 |
| ASGA0069511 | 15 | 43969428 | 0.8541 | 43835332 | 44063424 | ENSSSCG00000015797 | SORBS2 |
| H3GA0044304 | 15 | 43451810 | 0.8442 | 43427369 | 43628381 | ENSSSCG00000015789 | SNX25 |
| MARC0029600 | 4 | 97675204 | 0.7852 | 97674150 | 97704637 | ENSSSCG00000006490 | SMG5 |
| DRGA0005852 | 5 | 50100930 | 0.7889 | 50079369 | 50104219 | ENSSSCG00000000584 | SLCO1A2 |
| ASGA0021849 | 4 | 111894213 | 0.7957 | 111879347 | 111914029 | ENSSSCG00000006769 | SLC16A1 |
| H3GA0019890 | 7 | 10055550 | 0.8491 | 9985118 | 10185983 | ENSSSCG00000001055 | sirtuin-5 |
| DBNP0000897 | 19 | 84693332 | 0.8698 | 84691441 | 84697113 | ENSSSCG00000012547 | SERPINA7 |
| ASGA0018928 | 4 | 19939879 | 0.8002 | 19899321 | 20086556 | ENSSSCG00000006007 | SAMD12 |
| INRA0013758 | 4 | 37352337 | 0.9055 | 37325326 | 37479646 | ENSSSCG00000006069 | RGS22 |
| DRGA0005851 | 5 | 49978632 | 0.8489 | 49950143 | 50062422 | ENSSSCG00000000580 | RECQL |
| DRGA0001599 | 1 | 170658551 | 0.8305 | 170629212 | 170669040 | ENSSSCG00000004935 | PTPLAD1 |
| H3GA0004881 | 1 | 280040531 | 0.8031 | 280011355 | 280074547 | ENSSSCG00000005590 | Psmb7 |
| INRA0005593 | 1 | 199449597 | 0.8291 | 199376829 | 199488557 | ENSSSCG00000005095 | [PRKCQ](http://www.nextprot.org/entry/NX_Q04759) |
| H3GA0016074 | 5 | 21144007 | 0.7956 | 21142224 | 21156544 | ENSSSCG00000000408 | PRIM1 |
| DBUN0003725 | 19 | 65919177 | 0.9692 | 65918824 | 65919909 | ENSSSCG00000012454 | POU3F4 |
| INRA0056822 | 19 | 67608462 | 0.8698 | 67506761 | 67616334 | ENSSSCG00000012462 | POF1B |
| INRA0014351 | 4 | 63253372 | 0.8465 | 63239911 | 63263257 | ENSSSCG00000006172 | PI15 |
| DBNP0002253 | 19 | 57785871 | 0.8698 | 57722614 | 57824659 | ENSSSCG00000012411 | PHKA1 |
| M1GA0016423 | 12 | 20624642 | 0.8025 | 20622594 | 20637983 | ENSSSCG00000017502 | PGAP3 |
| DRGA0001807 | 1 | 194009274 | 0.8172 | 193971065 | 194179354 | ENSSSCG00000005061 | PELI2 |
| ISU10000372 | 2 | 26943600 | 0.878 | 26933326 | 26955190 | ENSSSCG00000013320 | PAX6 |
| BGIS0001442 | 19 | 62781681 | 0.8891 | 62767221 | 62782926 | ENSSSCG00000012446 | P2Y10 |
| ALGA0007312 | 1 | 194655153 | 0.8716 | 194652747 | 194656414 | ENSSSCG00000005063 | OTX2 |
| ALGA0099584 | 19 | 33824912 | 0.8019 | 33771381 | 33840748 | ENSSSCG00000012236 | OTC |
| H3GA0009550 | 3 | 49093213 | 0.7852 | 49022957 | 49126766 | ENSSSCG00000008171 | NPAS2 |
| H3GA0044826 | 15 | 102223112 | 0.8927 | 102184944 | 102223130 | ENSSSCG00000016127 | NDUFS1 |
| ASGA0064597 | 14 | 79831188 | 0.8285 | 79828336 | 79834081 | ENSSSCG00000010310 | NDST2 |
| INRA0049586 | 15 | 71918522 | 0.793 | 71824819 | 71923011 | ENSSSCG00000015941 | MYO15A |
| ALGA0027910 | 4 | 112186027 | 0.7861 | 112182903 | 112193989 | ENSSSCG00000006776 | MOV10 |
| INRA0014073 | 4 | 50345997 | 0.789 | 50225464 | 50382640 | ENSSSCG00000006132 | MMP16 |
| ALGA0007899 | 1 | 226138609 | 0.8281 | 226137877 | 226150391 | ENSSSCG00000005206 | MLANA |
| INRA0056759 | 19 | 56830694 | 0.8698 | 56826433 | 56849719 | ENSSSCG00000012396 | MED12 |
| MARC0019568 | 15 | 43832967 | 0.8541 | 43666471 | 43848616 | ENSSSCG00000015792 | LRP2BP |
| ASGA0004738 | 1 | 151144815 | 0.8507 | 151136184 | 151179791 | ENSSSCG00000004842 | KLF13 |
| DRGA0001958 | 1 | 226383550 | 0.8474 | 226296221 | 226407246 | ENSSSCG00000005208 | KIAA1432 |
| M1GA0005481 | 4 | 5143267 | 0.7983 | 5106428 | 5208907 | ENSSSCG00000005941 | KHDRBS3 |
| ALGA0007491 | 1 | 203995059 | 0.8083 | 203953036 | 204047668 | ENSSSCG00000005117 | KCNH5 |
| INRA0005996 | 1 | 226893907 | 0.8474 | 226776007 | 227058933 | ENSSSCG00000005215 | JAK2 |
| DRGA0015369 | 15 | 102120378 | 0.8927 | 102084002 | 102124382 | ENSSSCG00000016125 | INO80D |
| ALGA0069709 | 13 | 32593528 | 0.8287 | 32591562 | 32693479 | ENSSSCG00000011463 | IL17RD |
| MARC0041089 | 8 | 48090127 | 0.8195 | 48074809 | 48147152 | ENSSSCG00000008913 | IGFBP7 |
| INRA0057072 | 19 | 106705166 | 0.8488 | 106685807 | 106813960 | ENSSSCG00000012677 | HS6ST2 |
| INRA0056771 | 19 | 57673689 | 0.9515 | 57656340 | 57688846 | ENSSSCG00000012410 | HDAC8 |
| ALGA0037079 | 6 | 101899750 | 0.7994 | 101884549 | 101990156 | ENSSSCG00000003795 | GPR177 |
| ASGA0006040 | 1 | 241641945 | 0.8198 | 241341403 | 241643773 | ENSSSCG00000005285 | GNAQ |
| ASGA0096851 | 12 | 51733217 | 0.8395 | 51727140 | 51782021 | ENSSSCG00000017998 | GLP2R |
| ASGA0082757 | 1 | 288382387 | 0.8437 | 288375523 | 288396081 | ENSSSCG00000005737 | GFI1B |
| ALGA0085290 | 15 | 45611685 | 0.8394 | 45608605 | 45661032 | ENSSSCG00000015815 | FGFR |
| H3GA0024312 | 8 | 9634848 | 0.787 | 9627557 | 9647836 | ENSSSCG00000008746 | FAM184B |
| INRA0008331 | 2 | 16755336 | 0.8163 | 16664672 | 16805176 | ENSSSCG00000013281 | EXT2 |
| ALGA0007181 | 1 | 190705321 | 0.8784 | 190632580 | 190785319 | ENSSSCG00000005037 | ERO1L |
| CASI0001394 | 19 | 72470974 | 0.8698 | 72184642 | 72628052 | ENSSSCG00000012474 | DIAPH2 |
| ALGA0085908 | 15 | 71189033 | 0.8682 | 71172002 | 71194266 | ENSSSCG00000015930 | DHRS9 |
| INRA0056833 | 19 | 68463172 | 0.8698 | 68342129 | 68792554 | ENSSSCG00000012464 | DACH2 |
| INRA0014347 | 4 | 63121208 | 0.8045 | 63074087 | 63122685 | ENSSSCG00000006171 | CRISPLD1 |
| H3GA0004066 | 1 | 251504993 | 0.8474 | 251500327 | 251550387 | ENSSSCG00000005375 | CORO2A |
| BGIS0004952 | 8 | 32059576 | 0.8978 | 32051206 | 32061483 | ENSSSCG00000008811 | COMMD8 |
| MARC0003880 | 15 | 98550456 | 0.8083 | 98529731 | 98558003 | ENSSSCG00000016101 | C-FLIP |
| ASGA0006047 | 1 | 241828514 | 0.8415 | 241817636 | 241858211 | ENSSSCG00000005286 | CEP78 |
| ALGA0007919 | 1 | 226580040 | 0.8226 | 226573119 | 226592640 | ENSSSCG00000005211 | CD274 |
| DIAS0004461 | 13 | 56501538 | 0.8506 | 56454847 | 56515832 | ENSSSCG00000011578 | C3orf31 |
| INRA0049585 | 15 | 71423940 | 0.8026 | 71412980 | 71436255 | ENSSSCG00000015935 | C2orf77 |
| MARC0055277 | 13 | 138791556 | 0.7954 | 138776582 | 138792041 | ENSSSCG00000012037 | C21orf66 |
| BGIS0005780 | 1 | 196203548 | 0.8435 | 196157688 | 196203628 | ENSSSCG00000005071 | C14orf37 |
| DRGA0001810 | 1 | 194412280 | 0.8089 | 194402567 | 194509042 | ENSSSCG00000005062 | C14orf101 |
| MARC0099536 | 18 | 39394318 | 0.8308 | 39107251 | 39417923 | ENSSSCG00000016667 | BBS9 |
| ALGA0093995 | 17 | 26772789 | 0.8172 | 26761654 | 26806456 | ENSSSCG00000007087 | BANF2 |
| INRA0004031 | 1 | 127153803 | 0.8949 | 126930123 | 127186765 | ENSSSCG00000004646 | ATP8B4 |
| H3GA0003153 | 1 | 169208245 | 0.8507 | 169139942 | 169259478 | ENSSSCG00000004918 | ALPK2 |
| ALGA0100257 | 19 | 119399163 | 0.79 | 119284387 | 119584694 | ENSSSCG00000012730 | AFF2 |
