# Supplementary material for: A genome-wide scan for signatures of selection in Chinese indigenous and commercial pig breeds
Source: BMC Genet. 2014 Jan 15;15:7. doi: 10.1186/1471-2156-15-7 (PMC3898232; doi:10.1186/1471-2156-15-7)
Supplement: Additional file 2: Table S2 — Candidate genes under selection with SNPs in high Fst (CHN VS EURO). [file 1471-2156-15-7-S2.docx]

**Supplemental table 2: Candidate genes under selection with SNPs in high Fst** (**Commercial versus Chinese indigenous breeds).**

| SNP | Chr | Position | Fst | Gene Start (bp) | Gene End (bp) | Ensembl Gene ID | Within_Gene |
| --- | --- | --- | --- | --- | --- | --- | --- |
| INRA0003519 | 1 | 103707160 | 0.949 | 103637088 | 103714191 | ENSSSCG00000004522 | ME2 |
| CAIL0000072 | 1 | 103851428 | 0.949 | 103812352 | 103866422 | ENSSSCG00000004524 | SMAD4 |
| DRGA0001466 | 1 | 121936922 | 0.9859 | 121861605 | 122421065 | ENSSSCG00000004614 | UNC13C |
| ALGA0005765 | 1 | 124600282 | 0.9953 | 124590098 | 124603484 | ENSSSCG00000004624 | ERK3 |
| INRA0004031 | 1 | 127153803 | 0.9859 | 126930123 | 127186765 | ENSSSCG00000004646 | ATP8B4 |
| INRA0004312 | 1 | 143825053 | 0.9399 | 143734388 | 143908734 | ENSSSCG00000004803 | ACTC1 |
| ASGA0004639 | 1 | 145291290 | 0.9399 | 145050345 | 145349833 | ENSSSCG00000004812 | IGF1R |
| MARC0051707 | 1 | 151174698 | 0.9812 | 151136184 | 151179791 | ENSSSCG00000004842 | [KLF13](http://en.wikipedia.org/wiki/KLF13) |
| H3GA0003153 | 1 | 169208245 | 0.9812 | 169139942 | 169259478 | ENSSSCG00000004918 | ALPK2 |
| DRGA0001599 | 1 | 170658551 | 0.9756 | 170629212 | 170669040 | ENSSSCG00000004935 | PTPLAD1 |
| ALGA0007181 | 1 | 190705321 | 0.9859 | 190632580 | 190785319 | ENSSSCG00000005037 | ERO1A |
| DRGA0001807 | 1 | 194009274 | 0.9705 | 193971065 | 194179354 | ENSSSCG00000005061 | PELI2 |
| DRGA0001810 | 1 | 194412280 | 0.9582 | 194402567 | 194509042 | ENSSSCG00000005062 | C14orf101 |
| ALGA0007312 | 1 | 194655153 | 0.9906 | 194652747 | 194656414 | ENSSSCG00000005063 | OTX2 |
| INRA0005593 | 1 | 199449597 | 0.9706 | 199376829 | 199488557 | ENSSSCG00000005095 | [PRKCQ](http://www.ncbi.nlm.nih.gov/gene/5588) |
| MARC0045253 | 1 | 200697713 | 0.9437 | 200577776 | 200898360 | ENSSSCG00000005106 | NTRK3 |
| ALGA0007467 | 1 | 202969922 | 0.9859 | 202841045 | 203109516 | ENSSSCG00000005110 | SYNE2 |
| ALGA0007491 | 1 | 203995059 | 0.9601 | 203953036 | 204047668 | ENSSSCG00000005117 | KCNH5 |
| BGIS0007227 | 1 | 205148278 | 0.9859 | 205142406 | 205148310 | ENSSSCG00000005123 | TIE-2 |
| INRA0005811 | 1 | 214265959 | 0.944 | 214264832 | 214450132 | ENSSSCG00000005178 | CNTLN |
| ALGA0007899 | 1 | 226138609 | 0.9756 | 226137877 | 226150391 | ENSSSCG00000005206 | MLANA |
| DRGA0001958 | 1 | 226383550 | 0.9856 | 226296221 | 226407246 | ENSSSCG00000005208 | KIAA1432 |
| ALGA0007919 | 1 | 226580040 | 0.9695 | 226573119 | 226592640 | ENSSSCG00000005211 | PD-L1 |
| INRA0005996 | 1 | 226893907 | 0.9856 | 226776007 | 227058933 | ENSSSCG00000005215 | JAK2 |
| INRA0006204 | 1 | 234531067 | 0.9653 | 234286063 | 234571680 | ENSSSCG00000005257 | TRPM3 |
| ASGA0006040 | 1 | 241641945 | 0.944 | 241341403 | 241643773 | ENSSSCG00000005285 | GNAQ |
| ASGA0102957 | 1 | 241836023 | 0.9807 | 241817636 | 241858211 | ENSSSCG00000005286 | CEP78 |
| ALGA0008559 | 1 | 248156668 | 0.944 | 248152976 | 248174769 | ENSSSCG00000005341 | CLTA |
| ASGA0006152 | 1 | 248225395 | 0.9494 | 248180249 | 248286918 | ENSSSCG00000005342 | GNE |
| ALGA0008740 | 1 | 251478309 | 0.9905 | 251468742 | 251495122 | ENSSSCG00000005374 | TRIM14 |
| H3GA0004066 | 1 | 251504993 | 0.9806 | 251500327 | 251550387 | ENSSSCG00000005375 | CORO2A |
| H3GA0004881 | 1 | 280040531 | 0.9536 | 280011355 | 280074547 | ENSSSCG00000005590 | PSB7 |
| H3GA0009550 | 3 | 49093213 | 0.9493 | 49022957 | 49126766 | ENSSSCG00000008171 | NPAS2 |
| MARC0054644 | 3 | 53637819 | 0.9439 | 53578822 | 53658397 | ENSSSCG00000008226 | POLR1A |
| ASGA0019196 | 4 | 32089341 | 0.9454 | 31817202 | 32273033 | ENSSSCG00000006038 | ZFPM2 |
| ALGA0024569 | 4 | 35180442 | 0.9433 | 35169256 | 35201595 | ENSSSCG00000006058 | RRM2B |
| ALGA0025198 | 4 | 57797738 | 0.9906 | 57783815 | 57815361 | ENSSSCG00000006155 | ZBTB10 |
| INRA0014347 | 4 | 63121208 | 0.9654 | 63074087 | 63122685 | ENSSSCG00000006171 | CRISPLD1 |
| INRA0014351 | 4 | 63253372 | 0.9855 | 63239911 | 63263257 | ENSSSCG00000006172 | PI15 |
| H3GA0012850 | 4 | 66925043 | 0.9439 | 66905754 | 66936848 | ENSSSCG00000006193 | TRAM1 |
| ALGA0026258 | 4 | 86055343 | 0.9953 | 86049538 | 86082876 | ENSSSCG00000006301 | TIPRL |
| ASGA0021849 | 4 | 111894213 | 0.9491 | 111879347 | 111914029 | ENSSSCG00000006769 | MCT1 |
| ASGA0024488 | 5 | 10634072 | 0.944 | 10230975 | 10680952 | ENSSSCG00000000154 | SYN3 |
| ASGA0025072 | 5 | 20406304 | 0.9443 | 20325539 | 20460307 | ENSSSCG00000000372 | ERBB3 |
| CADI0000251 | 5 | 20734394 | 0.9443 | 20723188 | 20737703 | ENSSSCG00000000396 | STAT2 |
| H3GA0016069 | 5 | 20757310 | 0.9443 | 20751948 | 20776285 | ENSSSCG00000000399 | TIMELESS |
| H3GA0016074 | 5 | 21144007 | 0.9601 | 21142224 | 21156544 | ENSSSCG00000000408 | PRIM1 |
| ALGA0031742 | 5 | 35733150 | 0.9437 | 35506391 | 35909930 | ENSSSCG00000000515 | TRHDE |
| DRGA0005852 | 5 | 50100930 | 0.9492 | 50079369 | 50104219 | ENSSSCG00000000584 | SLCO1A2 |
| ALGA0031987 | 5 | 51990641 | 0.9437 | 51990478 | 52034702 | ENSSSCG00000000590 | PLCZ1 |
| ALGA0037079 | 6 | 101899750 | 0.9526 | 101884549 | 101990156 | ENSSSCG00000003795 | GPR177 |
| H3GA0019890 | 7 | 10055550 | 0.96 | 9985118 | 10185983 | ENSSSCG00000001055 | [SIRT5](http://www.ncbi.nlm.nih.gov/gene/23408) |
| ALGA0040847 | 7 | 42973791 | 0.9495 | 42868619 | 43071454 | ENSSSCG00000001635 | TAF8 |
| ASGA0033096 | 7 | 43494969 | 0.9495 | 43401505 | 43630342 | ENSSSCG00000001641 | UBR2 |
| ASGA0036455 | 7 | 123294718 | 0.9435 | 123234135 | 123348801 | ENSSSCG00000002463 | KIAA1409 |
| BGIS0004952 | 8 | 32059576 | 0.9906 | 32051206 | 32061483 | ENSSSCG00000008811 | COMMD8 |
| MARC0041089 | 8 | 48090127 | 0.9654 | 48074809 | 48147152 | ENSSSCG00000008913 | IGFBP7 |
| ASGA0083023 | 9 | 123421244 | 0.9508 | 123411172 | 123422059 | ENSSSCG00000015597 | NENF |
| M1GA0016423 | 12 | 20624642 | 0.9608 | 20622594 | 20637983 | ENSSSCG00000017502 | PGAP3 |
| ALGA0069709 | 13 | 32593528 | 0.972 | 32591562 | 32693479 | ENSSSCG00000011463 | IL17RD |
| MARC0055277 | 13 | 138791556 | 0.9462 | 138776582 | 138792041 | ENSSSCG00000012037 | C21orf66 |
| ASGA0059913 | 13 | 140043340 | 0.9462 | 140021472 | 140061224 | ENSSSCG00000012051 | RUNX1 |
| INRA0041627 | 13 | 141013546 | 0.9423 | 141003285 | 141123174 | ENSSSCG00000012062 | TTC3 |
| ASGA0064597 | 14 | 79831188 | 0.9706 | 79828336 | 79834081 | ENSSSCG00000010310 | NDST2 |
| DRGA0015369 | 15 | 102120378 | 0.9667 | 102084002 | 102124382 | ENSSSCG00000016125 | INO80D |
| H3GA0044826 | 15 | 102223112 | 0.9667 | 102184944 | 102223130 | ENSSSCG00000016127 | NDUFS1 |
| ALGA0093995 | 17 | 26772789 | 0.9493 | 26761654 | 26806456 | ENSSSCG00000007087 | BANF2 |
| MARC0099536 | 19 | 39394318 | 0.9758 | 39107251 | 39417923 | ENSSSCG00000016667 | BBS9 |
| ALGA0099584 | 19 | 33824912 | 0.9653 | 33771381 | 33840748 | ENSSSCG00000012236 | OTC |
| INRA0061544 | 19 | 41912527 | 0.9545 | 41800482 | 41973581 | ENSSSCG00000012271 | RBM10 |
| INRA0056742 | 19 | 50337998 | 0.9439 | 50306207 | 50393778 | ENSSSCG00000012362 | ARHGEF9 |
| INRA0056759 | 19 | 56830694 | 0.9953 | 56826433 | 56849719 | ENSSSCG00000012396 | MED12 |
| INRA0056771 | 19 | 57673689 | 1 | 57656340 | 57688846 | ENSSSCG00000012410 | HDAC8 |
| DBNP0002253 | 19 | 57785871 | 0.9953 | 57722614 | 57824659 | ENSSSCG00000012411 | PHKA1 |
| BGIS0001442 | 19 | 62781681 | 1 | 62767221 | 62782926 | ENSSSCG00000012446 | P2Y |
| DBUN0003725 | 19 | 65919177 | 1 | 65918824 | 65919909 | ENSSSCG00000012454 | POU4 |
| INRA0056822 | 19 | 67608462 | 0.9953 | 67506761 | 67616334 | ENSSSCG00000012462 | POF1B |
| INRA0056836 | 19 | 68665655 | 0.9953 | 68342129 | 68792554 | ENSSSCG00000012464 | DACH2 |
| CASI0001394 | 19 | 72470974 | 0.9953 | 72184642 | 72628052 | ENSSSCG00000012474 | DIAPH2 |
| MARC0018104 | 19 | 75615013 | 0.9806 | 75611723 | 75635367 | ENSSSCG00000012480 | TNMD |
| ALGA0099836 | 19 | 84403863 | 0.9439 | 84381760 | 84449091 | ENSSSCG00000012545 | IL1RAPL-2 |
| DBNP0000897 | 19 | 84693332 | 0.9953 | 84691441 | 84697113 | ENSSSCG00000012547 | TBG |
| ALGA0099994 | 19 | 97354692 | 0.9438 | 97286919 | 97425607 | ENSSSCG00000012634 | DOCK11 |
| INRA0057072 | 19 | 106705166 | 0.9812 | 106685807 | 106813960 | ENSSSCG00000012677 | HS6ST2 |
