# Supplementary material for: A genome-wide scan for signatures of selection in Chinese indigenous and commercial pig breeds
Source: BMC Genet. 2014 Jan 15;15:7. doi: 10.1186/1471-2156-15-7 (PMC3898232; doi:10.1186/1471-2156-15-7)
Supplement: Additional file 3: Table S3 — Candidate genes under selection with SNPs in high Fst (Northern VS Southern). [file 1471-2156-15-7-S3.docx]

**Supplemental table 3: Candidate genes under selection with SNPs in high Fst (Northern versus Southern Chinese indigenous breeds).**

| SNP | Chr | Position | Fst | Gene Start (bp) | Gene End (bp) | Ensembl Gene ID | Within_Gene |
| --- | --- | --- | --- | --- | --- | --- | --- |
| ASGA0090411 | 1 | 15415763 | 0.9614 | 15186680 | 15450209 | ENSSSCG00000004083 | ESR1 |
| ALGA0002593 | 1 | 36595352 | 0.9291 | 36404866 | 36761816 | ENSSSCG00000004209 | PTPRK |
| H3GA0002769 | 1 | 137373068 | 0.9714 | 137362220 | 137377017 | ENSSSCG00000004774 | IVD |
| CASI0003490 | 1 | 252371845 | 0.8757 | 252356422 | 252431941 | ENSSSCG00000005380 | COL15A1 |
| ALGA0008866 | 1 | 254393393 | 0.951 | 254350768 | 254688760 | ENSSSCG00000005393 | LPPR1 |
| INRA0006961 | 1 | 260581780 | 0.9023 | 260561463 | 260613668 | ENSSSCG00000005435 | ZNF462 |
| DBWU0000818 | 2 | 7708228 | 0.8747 | 7664992 | 7709096 | ENSSSCG00000013065 | ASRGL1 |
| ASGA0008896 | 2 | 7880186 | 0.8574 | 7867245 | 7887061 | ENSSSCG00000013073 | FADS3 |
| ASGA0008918 | 2 | 8023620 | 0.863 | 8014755 | 8047559 | ENSSSCG00000013078 | C11orf9 |
| ALGA0011799 | 2 | 8406128 | 0.8438 | 8394734 | 8412259 | ENSSSCG00000013083 | CPSF7 |
| DBMA0000048 | 2 | 56360865 | 0.8899 | 56360272 | 56363181 | ENSSSCG00000013752 | STX10 |
| ALGA0015239 | 2 | 105676179 | 0.9812 | 105636958 | 105764708 | ENSSSCG00000014207 | APC |
| DIAS0004466 | 2 | 127217533 | 0.8763 | 127201864 | 127218283 | ENSSSCG00000014327 | BRD8 |
| ASGA0083885 | 2 | 134563166 | 0.8703 | 134553641 | 134609923 | ENSSSCG00000014420 | JAKMIP2 |
| M1GA0003510 | 2 | 136374416 | 0.8937 | 136367690 | 136425357 | ENSSSCG00000014438 | PDE6A |
| MARC0043512 | 3 | 4082727 | 0.8969 | 4068724 | 4094386 | ENSSSCG00000007590 | PMS2 |
| ASGA0101753 | 3 | 5635189 | 0.8631 | 5624909 | 5832347 | ENSSSCG00000007615 | CPSF4 |
| ALGA0018472 | 3 | 30039994 | 1 | 29888769 | 30048895 | ENSSSCG00000007909 | GABT |
| DRGA0004022 | 3 | 66630379 | 0.9614 | 66585291 | 66642915 | ENSSSCG00000008335 | GMCL1 |
| ASGA0015389 | 3 | 87764089 | 0.8561 | 87746073 | 87930939 | ENSSSCG00000008444 | PRKCE |
| H3GA0010886 | 3 | 115052966 | 0.9296 | 115035691 | 115056815 | ENSSSCG00000008623 | TRIB2 |
| MARC0020606 | 4 | 36711451 | 0.8553 | 36694058 | 36742254 | ENSSSCG00000006064 | SNX31 |
| INRA0013758 | 4 | 37352337 | 1 | 37325326 | 37479646 | ENSSSCG00000006069 | RGS22 |
| H3GA0012835 | 4 | 65671657 | 0.9486 | 65646472 | 65696166 | ENSSSCG00000006186 | TRPA1 |
| ALGA0026338 | 4 | 88510508 | 0.9365 | 88506142 | 88531579 | ENSSSCG00000006325 | [MGST3](http://www.genecards.org/cgi-bin/carddisp.pl?gene=MGST3) |
| ASGA0021815 | 4 | 111458850 | 0.8949 | 111240276 | 111477420 | ENSSSCG00000006767 | MAGI3 |
| ASGA0023344 | 4 | 132203028 | 0.8461 | 132178270 | 132215083 | ENSSSCG00000006926 | GTF2B |
| DIAS0000180 | 5 | 4929364 | 0.9372 | 4916295 | 4970998 | ENSSSCG00000000082 | CACNA1I |
| ALGA0030674 | 5 | 11819369 | 0.949 | 11800623 | 11894144 | ENSSSCG00000000168 | RFX4 |
| H3GA0015948 | 5 | 16842514 | 0.9713 | 16840647 | 16849864 | ENSSSCG00000000254 | TENC1 |
| ASGA0025136 | 5 | 23110344 | 1 | 23056590 | 23129888 | ENSSSCG00000000455 | LRIG3 |
| H3GA0016981 | 5 | 78318631 | 0.8441 | 78226281 | 78452363 | ENSSSCG00000000869 | UTP20 |
| ALGA0033767 | 5 | 90356489 | 0.849 | 90339464 | 90386163 | ENSSSCG00000000927 | TMTC3 |
| DIAS0001800 | 6 | 12214973 | 0.9182 | 12213792 | 12334637 | ENSSSCG00000002755 | NFAT5 |
| ASGA0097426 | 6 | 56380402 | 0.9405 | 56366772 | 56390456 | ENSSSCG00000003539 | GRHL3 |
| ALGA0035889 | 6 | 60707033 | 0.9015 | 60701999 | 60726958 | ENSSSCG00000003606 | SPOCD1 |
| ALGA0103876 | 6 | 74143978 | 0.8952 | 73905801 | 74181775 | ENSSSCG00000003709 | LAMA3 |
| INRA0026529 | 7 | 81308240 | 0.8688 | 81297178 | 81313143 | ENSSSCG00000001988 | ADCY4 |
| ALGA0049130 | 8 | 94034689 | 0.9405 | 93935642 | 94260375 | ENSSSCG00000009125 | ANK2 |
| ALGA0055709 | 9 | 124812714 | 0.9372 | 124688872 | 124962380 | ENSSSCG00000015607 | HHAT |
| H3GA0028979 | 10 | 3564686 | 0.9549 | 3488327 | 3823194 | ENSSSCG00000010809 | FAM5C |
| H3GA0029488 | 10 | 17802122 | 0.9047 | 17787840 | 17979746 | ENSSSCG00000010879 | KIF26B |
| ASGA0094215 | 10 | 24987426 | 0.9194 | 24833795 | 25022842 | ENSSSCG00000010928 | KDM5B |
| DIAS0000707 | 10 | 24985343 | 0.8443 | 24955012 | 24986671 | ENSSSCG00000010931 | KLHL12 |
| ALGA0108169 | 11 | 67498853 | 0.9025 | 67466727 | 67545142 | ENSSSCG00000009505 | MBNL2 |
| MARC0027540 | 13 | 21884909 | 0.8463 | 21872535 | 21944268 | ENSSSCG00000011280 | VIPR1 |
| ALGA0069288 | 13 | 23964485 | 0.9532 | 23900998 | 23965720 | ENSSSCG00000011309 | KIF15 |
| MARC0005592 | 13 | 40025759 | 0.9812 | 40001091 | 40242551 | ENSSSCG00000011499 | LRIG1 |
| ASGA0099677 | 13 | 40859421 | 0.8887 | 40717893 | 41010484 | ENSSSCG00000011501 | SCS |
| ASGA0058640 | 13 | 78817766 | 0.9067 | 78782572 | 78821617 | ENSSSCG00000011731 | SMC4 |
| ASGA0062713 | 14 | 35463058 | 0.8574 | 35461076 | 35495959 | ENSSSCG00000009857 | NOS1 |
| ALGA0077360 | 14 | 45826379 | 0.8568 | 45775702 | 45838506 | ENSSSCG00000009967 | PITPNB |
| DIAS0002579 | 14 | 49681978 | 0.9714 | 49670401 | 49703815 | ENSSSCG00000010039 | SGLT2 |
| ALGA0079765 | 14 | 93521313 | 0.9296 | 93472678 | 93560161 | ENSSSCG00000010385 | C10orf72 |
| ASGA0065280 | 14 | 94708400 | 1 | 94616209 | 94733974 | ENSSSCG00000010402 | ANUBL1 |
| ASGA0065469 | 14 | 100305987 | 0.8529 | 100197944 | 100670231 | ENSSSCG00000010426 | PCDH15 |
| M1GA0019170 | 14 | 129782300 | 0.8571 | 129765308 | 129799912 | ENSSSCG00000010639 | HABP2 |
| ALGA0081830 | 14 | 130350968 | 0.8571 | 130342093 | 130380210 | ENSSSCG00000010648 | C10orf118 |
| ASGA0066748 | 14 | 131185261 | 0.8949 | 131145416 | 131187908 | ENSSSCG00000010653 | TRUB1 |
| H3GA0044304 | 15 | 43451810 | 0.9489 | 43427369 | 43628381 | ENSSSCG00000015789 | SNX25 |
| MARC0019568 | 15 | 43832967 | 0.9511 | 43666471 | 43848616 | ENSSSCG00000015792 | LRP2BP |
| ASGA0069511 | 15 | 43969428 | 0.9511 | 43835332 | 44063424 | ENSSSCG00000015797 | SORBS2 |
| ALGA0085290 | 15 | 45611685 | 1 | 45608605 | 45661032 | ENSSSCG00000015815 | FGFR |
| ASGA0069559 | 15 | 45768991 | 0.9401 | 45762923 | 45773350 | ENSSSCG00000015817 | BAG4 |
| ASGA0069921 | 15 | 73167394 | 0.8521 | 73134121 | 73279312 | ENSSSCG00000015952 | HAT1 |
| DRGA0015447 | 15 | 107376829 | 0.8496 | 107147960 | 107599764 | ENSSSCG00000016160 | ERBB4 |
| ASGA0106268 | 15 | 124784965 | 0.8621 | 124737803 | 124839293 | ENSSSCG00000016280 | COPS7B |
| MARC0004304 | 17 | 25554698 | 0.906 | 25364030 | 25629924 | ENSSSCG00000007080 | KIF16B |
| ASGA0076903 | 17 | 41631478 | 0.8951 | 41631092 | 41649356 | ENSSSCG00000007315 | C20orf4 |
| ASGA0077527 | 17 | 53616061 | 0.869 | 53559298 | 53649678 | ENSSSCG00000007466 | SLC9A8 |
| MARC0027974 | 17 | 55038439 | 0.9211 | 54981839 | 55084049 | ENSSSCG00000007477 | NFAT |
| DBWU0000937 | 18 | 10718173 | 0.9405 | 10629256 | 10718361 | ENSSSCG00000016522 | PTN |
| H3GA0050863 | 18 | 41160349 | 0.8625 | 41133118 | 41162880 | ENSSSCG00000016681 | C7orf41 |
| H3GA0051007 | 18 | 44628244 | 0.9511 | 44439045 | 44669864 | ENSSSCG00000016708 | SKAP2 |
| MARC0111499 | 19 | 5764387 | 0.8932 | 5754117 | 5861297 | ENSSSCG00000012108 | WWC3 |
| ASGA0080749 | 19 | 6946218 | 0.8947 | 6754432 | 7036677 | ENSSSCG00000012112 | ARHGAP6 |
| CAHM0000185 | 19 | 36694003 | 0.8822 | 36683848 | 36697312 | ENSSSCG00000012252 | Q8HZ78 |
| H3GA0052592 | 19 | 41973203 | 0.8807 | 41800482 | 41973581 | ENSSSCG00000012271 | RBM10 |
| ALGA0100211 | 19 | 112243524 | 1 | 112232954 | 112342243 | ENSSSCG00000012712 | MCF2 |
