# Supplementary figures and images for: A genome-wide scan for signatures of selection in Chinese indigenous and commercial pig breeds
Source: BMC Genet. 2014 Jan 15;15:7. doi: 10.1186/1471-2156-15-7 (PMC3898232; doi:10.1186/1471-2156-15-7)

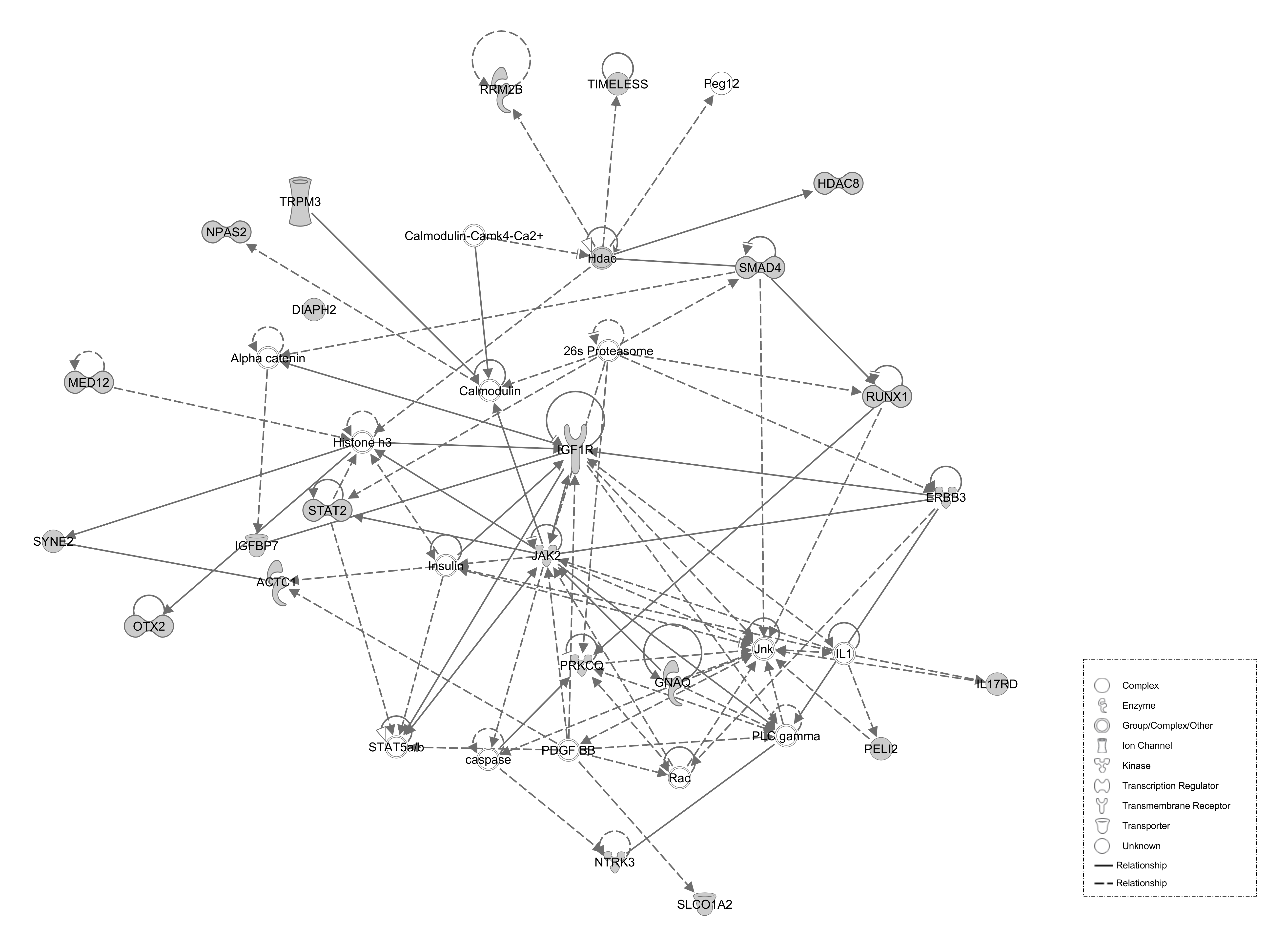

Supplement: Additional file 4: Figure S1 — Representation of the gene network group CHN VS EURO. Symbols corresponding to genes under selection are colored in grey. [file 1471-2156-15-7-S4.tiff]

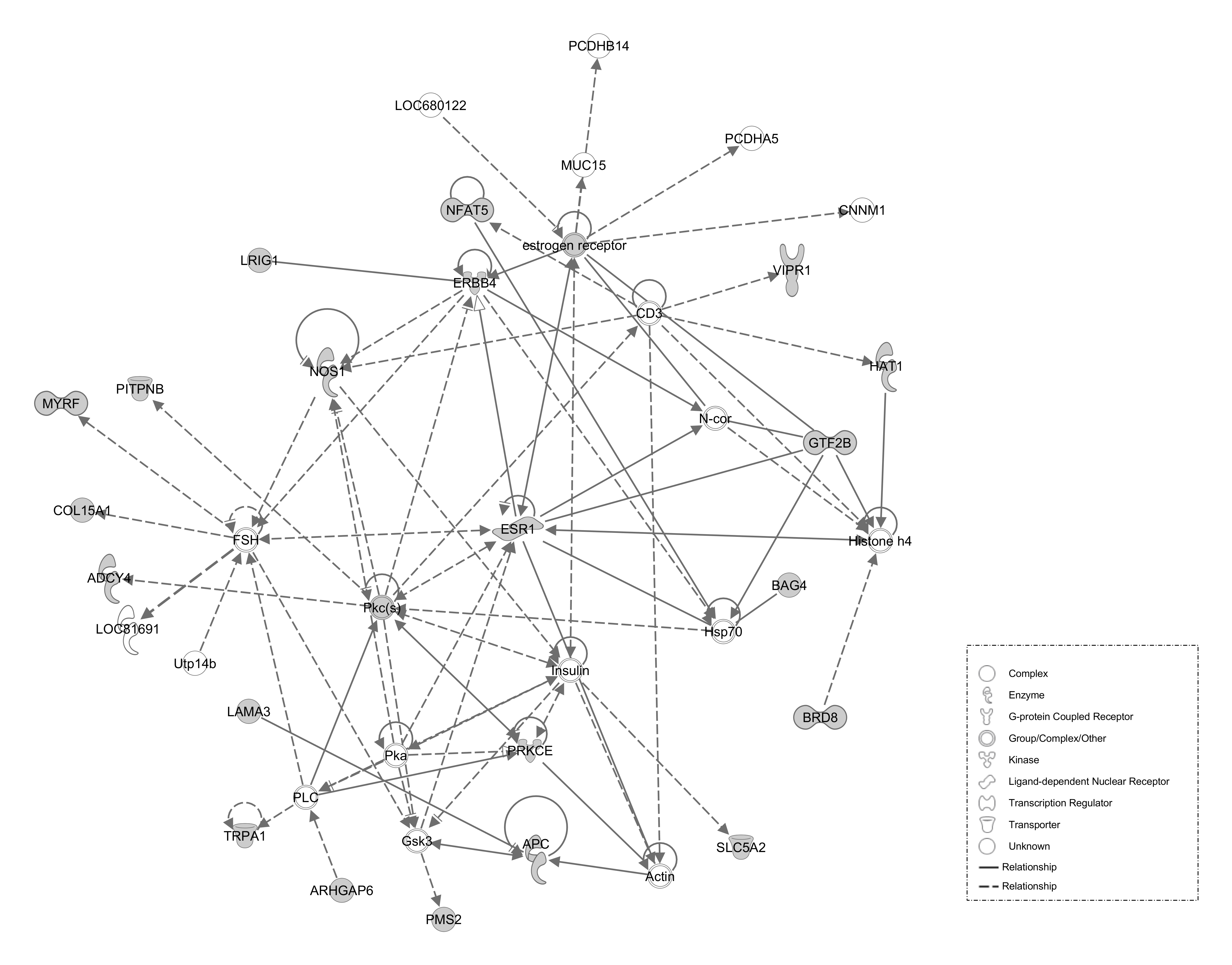

Supplement: Additional file 5: Figure S2 — Representation of the gene network group Northern VS Southern. Symbols corresponding to genes under selection are colored in grey. [file 1471-2156-15-7-S5.tiff]
